# Integrated Multi-Omics Reveals Mechanism of Adventitious Buds Regeneration in In Vitro Cultures of *Cinnamomum parthenoxylon*

**DOI:** 10.3390/plants14192945

**Published:** 2025-09-23

**Authors:** Chenglin Luo, Xin Qiao, Xiaoying Dai, Yuntong Zhang, Xinliang Liu, Yanfang Wu

**Affiliations:** 1 Co-Innovation Center for Sustainable Forestry in Southern China, Nanjing Forestry University, 159 Longpan Road, Nanjing 210037, China; luochenglin@njfu.edu.cn (C.L.); 15839183042@njfu.edu.cn (X.Q.); 2 Camphor Engineering and Technology Research Centre of National Forestry and Grassland Administration, Jiangxi Academy of Forestry, Nanchang 330032, China; dxy3842678@sina.com (X.D.); canon534534@163.com (Y.Z.)

**Keywords:** *Cinnamomum parthenoxylon*, in vitro regeneration, callus, morphology, physiological indices, RNA-seq

## Abstract

A pluripotent callus is central to genetic transformation in *Cinnamomum parthenoxylon*; however, the molecular and cellular mechanisms regulating callus formation and subsequent differentiation remain unelucidated, hindering progress in its genetic improvement. This study systematically investigated the dynamic changes during the in vitro regeneration of *C. parthenoxylon* through morphological observations, physiological assays, and transcriptomic analyses, while comparing differences in callus formation under varying induction conditions to elucidate the mechanism of its high-efficiency regeneration. The results showed that the formation of a pluripotent callus is a critical step in *C. parthenoxylon* regeneration, characterized by the presence of highly proliferative cell zones. Compared to an ordinary callus (P3C), a pluripotent callus (P3) exhibited higher activities of polyphenol oxidase (PPO) and indole-3-acetic acid oxidase (IAAO), as well as elevated levels of zeatin riboside (ZR) and abscisic acid (ABA). In contrast, P3 showed lower levels of soluble sugars, soluble proteins, malondialdehyde (MDA), indole-3-acetic acid (IAA), and gibberellins (GA), a reduced IAA/ZR ratio, and diminished peroxidase (POD) activity. Weighted gene co-expression network analysis (WGCNA) identified 27 hub transcription factors (TFs) strongly associated with IAA/ZR, primarily from the ERF, bHLH, MYB, WRKY, and C3H families. Gene Ontology (GO) and the Kyoto Encyclopedia of Genes and Genomes (KEGG) pathway enrichment analyses revealed the significant enrichment of differentially expressed genes (DEGs) related to plant hormone signal transduction and cell wall metabolism during pluripotent callus acquisition. Further investigations revealed that five genes encoding a putative indole-3-acetic acid-amido synthetase GH3.1, protein TIFY 10A, a two-component response regulator ARR2-like isoform X2, and xyloglucan endotransglucosylase/hydrolase, likely promoting callus pluripotency by modulating plant hormone signaling and cell wall metabolism, thereby enhancing in vitro regeneration in *C. parthenoxylon*. In summary, this study provides critical insights into the molecular mechanisms of *C. parthenoxylon* regeneration and offers valuable germplasm resources for establishing an efficient and stable genetic transformation system via tissue culture.

## 1. Introduction

*Cinnamomum parthenoxylon*, a tree species endemic to southern China, is highly valued for its applications in essential oil production and landscaping, making it an economically significant resource. Its leaves contain abundant essential oils composed primarily of monoterpenes and sesquiterpenes, including key components such as linalool, camphor, and citral [1]. These essential oils serve as valuable natural flavorings and chemical raw materials, with extensive applications in food, pharmaceutical, and cosmetic industries. Current research indicates that linalool interacts with multiple molecular targets in humans, exhibiting diverse therapeutic effects that make it a promising candidate for chronic disease treatment [2]. Beyond its use as a flavoring agent, citral demonstrates effective food preservation properties. For instance, active packaging incorporating citral has been shown to significantly inhibit the respiration rate, weight loss, and microbial growth in postharvest strawberries, thereby extending the shelf life [3]. The biosynthesis and accumulation of essential oils represent dynamic processes closely associated with the genes’ expression patterns. Recent studies have extensively investigated terpenoid biosynthesis-related genes across various plant species. In Lauraceae plants, which are particularly rich in terpenoids, several key genes involved in terpenoid biosynthesis have been successfully identified and functionally characterized [4,5]. However, the absence of an efficient genetic transformation system has the limited functional validation of candidate genes, consequently impeding progress in improving both the quality and yield of superior *C. parthenoxylon* cultivars.

Plant genetic transformation serves as an essential tool for plant genetic engineering and modern molecular breeding. Its transformation efficiency is primarily dependent on two critical steps: (1) the transfer and expression of foreign DNA in host cells and (2) the ability to develop fertile plants from transformed cells. For most species, regeneration represents the fundamental step in obtaining transgenic plants, a process that relies on pluripotent callus formation. A pluripotent callus develops through cellular redifferentiation [6], predominantly via the lateral root developmental pathway, exhibiting structural similarities to root apical meristems [7]. Callus pluripotency is regulated by both endogenous and exogenous factors involving cellular fate transition, with root primordium-like characteristics serving as the cellular basis of callus pluripotency [8]. Phytohormones, particularly auxins and cytokinins, play pivotal roles in in vitro plant regeneration. Research demonstrates that the auxin-to-cytokinin ratio determines the organogenesis direction—high ratios promote adventitious root formation while low ratios induce shoot regeneration [9]. Auxins are considered as master regulators of the cellular fate transition, inducing the expression of *PLT3*, *PLT5*, and *PLT7*. These transcription factors subsequently activate root meristem signature genes (*PLT1* and *PLT2*) and shoot regeneration markers (*CUC1* and *CUC2*), thereby conferring the shoot regeneration capacity to the callus [10]. Cytokinins represent essential factors for shoot induction, where type-B ARRs (key components of cytokinin signaling) directly bind to and activate the *WUS* promoter [11]. *WUS* serves as a crucial regulator of de novo shoot regeneration and maintains shoot apical meristem (SAM) stem cell activity [12].

The in vitro regeneration process is governed by numerous developmental regulators, whose overexpression offers solutions for species with low regeneration frequencies or genotype-dependent limitations. For instance, the overexpression of *MdBBM1* significantly enhances the transformation and regeneration efficiency in apples, producing healthy transgenic plants suitable for secondary transformation studies [13]. Genes such as *GRF5* and *PLT5* have proven effective in overcoming genotype-dependent transformation barriers across various plant species [14,15]. Although the regeneration system for *C. parthenoxylon* based on callus formation has been established [16], its application remains constrained due to strong genotype dependence, low reproducibility, and a progressive decline in the differentiation capacity during subculture. These constraints pose major obstacles to the development of efficient transformation protocols. To establish a robust regeneration system with improved shoot regeneration and transformation rates, this study systematically investigated morphological characteristics, histological changes, endogenous substances, and gene expression patterns during stem segment regeneration in *C. parthenoxylon*. In this study, the regeneration process was systematically investigated. Furthermore, key developmental stages were selected to compare the differences between a pluripotent callus and ordinary callus at various levels. Through a comparative analysis of ordinary versus pluripotent calluses, we identified cell clusters with differentiation potential within pluripotent callus tissues, whose size and area may serve as indicative markers for optimizing callus induction conditions in subsequent studies. In the transcriptomic analysis, we first identified key transcription factors (TFs) strongly associated with auxin and cytokinin by WGCNA. Subsequently, we clustered differentially expressed genes (DEGs) and focused on pathways and functional enrichments of those highly expressed during the acquisition of callus pluripotency. Finally, by analyzing the correlation between TFs and genes within critical pathways, we pinpointed candidate genes linked to callus pluripotency. The scientific and rational selection of materials and analytical methods ensured the reliability of the research findings. In subsequent studies, these key genes will be introduced into both degenerated and ordinary callus tissues, not only to validate their functions, but also to significantly enhance the pluripotency and genetic transformation efficiency of the callus. This study provides fundamental insights into the molecular mechanisms governing *C. parthenoxylon* regeneration and valuable genetic resources for future studies aiming to enhance the regeneration efficiency through developmental regulator transformation.

## 2. Results

### 2.1. Morphological and Histocytological Characteristics of Regeneration

**Figure 1:**
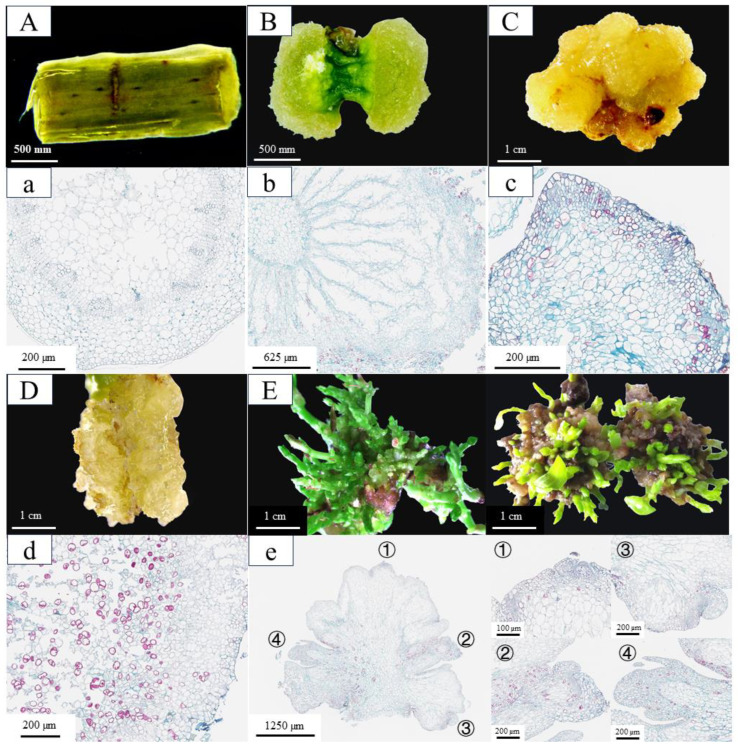
The phenotypic and histological characteristics of in vitro regeneration in *C. parthenoxylon*. (**A**,**a**) Morphological (**A**) and histological (**a**) observations of explants prior to induction (P1). (**B**,**b**) Morphological (**B**) and histological (**b**) features after 20 days of culture under 6-BA and 2,4-D treatment (P2). (**C**,**c**) Morphological (**C**) and histological (**c**) characteristics following 35 days of 6-BA and 2,4-D treatment (P3). (**D**,**d**) Morphological (**D**) and histological (**d**) analysis after 35 days of 6-BA and NAA treatment (P3C). (**E**,**e**) Morphological (**E**) and histological (**e**) evaluation after 10 days of adventitious bud induction with 6-BA, TDZ, and NAA treatment (P4). The right panel shows an enlarged view of the corresponding area designated by the box in the left panel in (**e**).

Under the synergistic action of 6-BA and 2,4-D, parenchyma cells adjacent to the incision sites of stem segments underwent substantial morphological alterations (Figure 1). By day 20 of culture, pronounced swelling was observed at both ends of the stem segments, resulting from extensive cell division. This led to the formation of dense, green callus tissues that entirely concealed the original epidermal structure (Figure 1B,b). By day 35, the callus had entered a rapid proliferation phase, during which its color shifted from green to yellow and its texture became increasingly compact (Figure 1C). Observations from paraffin sections showed distinct cell clusters at the callus periphery, characterized by a small cell size, high nuclear-to-cytoplasmic ratios, and tightly packed arrangements. These morphological features are indicative of a pluripotent callus with high differentiation potential (Figure 1c). When transferred to SIM (MS + 1.0 mg/L 6-BA + 0.8 mg/L TDZ + 0.05 mg/L NAA), these calluses produced numerous adventitious buds within just 10 days (Figure 1E). In contrast, calluses induced by 6-BA and NAA exhibiting distinct morphological differences, including a high water content, friable texture (Figure 1D), and loose cellular arrangement composed of large-cell-lacking nucleoli and showing extensive cellular disintegration (Figure 1d). This kind of callus completely lacked pluripotent characteristics and failed to differentiate adventitious buds when transferred to SIM. These comparative results demonstrate that the formation of pluripotent calluses is a critical determinant for successful in vitro regeneration in *C. parthenoxylon*.

### 2.2. Dynamic Changes in Endogenous Nutrients, Enzymes, and Hormones During Adventitious Bud Formation

**Figure 2:**
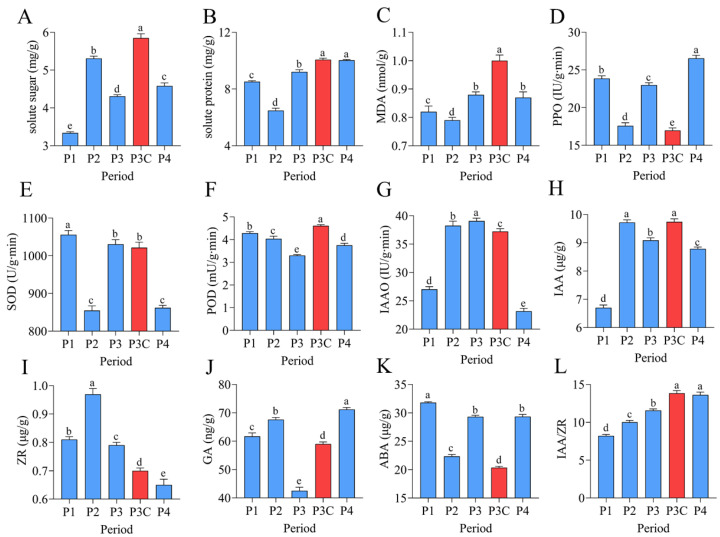
Dynamic changes in endogenous nutrients, enzyme activities, and phytohormones during adventitious bud formation. (**A**) Soluble sugars. (**B**) Soluble proteins. (**C**) MDA. (**D**) PPO. (**E**) SOD. (**F**) POD. (**G**) IAAO. (**H**) IAA. (**I**) ZR. (**J**) GA. (**K**) ABA. (**L**) IAA/ZR ratio. Data are presented as mean ± SD (*n* = 6). Different lowercase letters (a–e) indicate significant differences in physiological indices among samples at the 0.05 level, as determined by one-way ANOVA and LSD multiple comparison test.

The in vitro regeneration of *C. parthenoxylon* was accompanied by marked dynamic changes in endogenous nutrient levels, enzyme activities, and phytohormone contents (Figure 2). A statistical analysis revealed that all measured parameters exhibited significant differences (*p* < 0.05) across various developmental stages. During the callus formation phase (P1–P2), the soluble sugar content increased markedly while the soluble protein content decreased significantly, both reaching their extreme values at P2. The transition to the pluripotent callus acquisition phase (P2–P3) was characterized by a rapid decline in soluble sugars and a concurrent increase in soluble proteins. The malondialdehyde (MDA) content, along with the activities of polyphenol oxidase (PPO) and superoxide dismutase (SOD), decreased significantly during callus formation, but increased notably during pluripotent callus development. In contrast, peroxidase (POD) and indoleacetic acid oxidase (IAAO) activities showed opposite trends. The POD activity decreased initially, reaching its lowest level at stage P3, and then increased, while the IAAO activity rose first, peaked at P3, and subsequently declined. The phytohormone analysis revealed a rapid accumulation of indole-3-acetic acid (IAA) and zeatin riboside (ZR) during callus formation, with both reaching peak levels at stage P2 before gradually declining. In contrast, the gibberellin (GA) content increased markedly during callus formation, then dropped sharply as pluripotent calluses developed, reaching a minimum at stage P3, followed by a rebound in the subsequent differentiation phase. The abscisic acid (ABA) levels exhibited a characteristic pattern of an initial decrease, followed by an increase, and subsequent stabilization, whereas the IAA/ZR ratio increased progressively throughout the regeneration process. A comparative analysis between a pluripotent callus (P3) and ordinary callus (P3C) revealed significant physiological differences. The pluripotent callus exhibited higher activities of PPO and IAAO, as well as elevated ZR and ABA contents. In contrast, it contained lower levels of soluble sugars, soluble proteins, MDA, IAA, GA, and POD activity, along with a reduced IAA/ZR ratio. These findings indicate that successful adventitious bud formation depends on the precise coordination of the nutrient metabolism, enzymatic activities, and hormonal balance across distinct stages of regeneration.

### 2.3. RNA Sequencing Analysis During Adventitious Bud Formation

**Figure 3:**
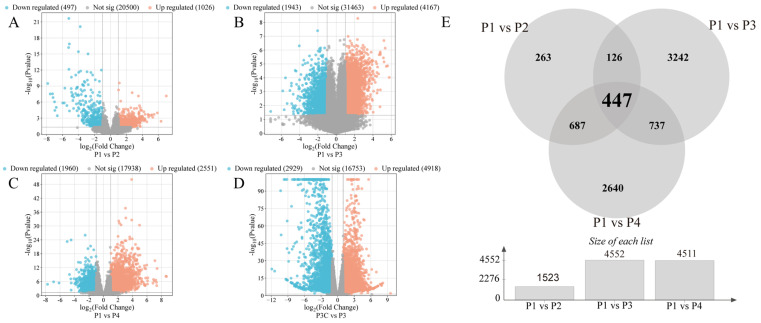
Statistics of differentially expressed genes (DEGs). (**A**–**D**) Volcano plots of DEGs in different comparisons. (**A**) P1 vs. P2. (**B**) P1 vs. P3. (**C**) P1 vs. P4. (**D**) P3C vs. P3. (**E**) Venn diagram of DEGs in different comparisons.

To elucidate the molecular mechanisms underlying in vitro regeneration in *C. parthenoxylon*, we conducted comprehensive transcriptome sequencing across key developmental stages. The high reproducibility of our data was confirmed by strong Pearson correlation coefficients among biological replicates (Table S2) and consistent expression patterns between the RNA-seq and qRT-PCR results (Figure S1). Differential expression analysis using pre-inoculation samples (P1) as controls identified 1523 DEGs (1026 upregulated/497 downregulated) in P1 vs. P2, 6110 DEGs (4167/1943) in P1 vs. P3, and 4471 DEGs (2551/1960) in P1 vs. P4 comparisons (Figure 3A–E). All comparisons showed a greater number of upregulated than downregulated genes. Throughout the entire regeneration process, a total of 7917 differentially expressed genes (DEGs) were identified. Among these, 447 genes were consistently differentially expressed across all comparisons and are likely to represent core regulators of regeneration (Figure 3E). The P1 vs. P3 comparison exhibited the most extensive transcriptional changes, accounting for 77% of all DEGs, highlighting this stage as the most dynamically regulated phase. Importantly, a comparison between an ordinary callus (P3C) and pluripotent callus (P3) identified 7847 DEGs (Figure 3C). Among these, 1662 and 2697 overlapped with DEGs from the P1 vs. P3 and P1 vs. P4 comparisons, respectively (Figure S2). This result demonstrates that the acquisition of pluripotency represents a critical regulatory transition during *C. parthenoxylon* regeneration, marked by extensive transcriptional reprogramming.

### 2.4. Identification of TFs Co-Expression Modules by WGCNA

**Figure 4:**
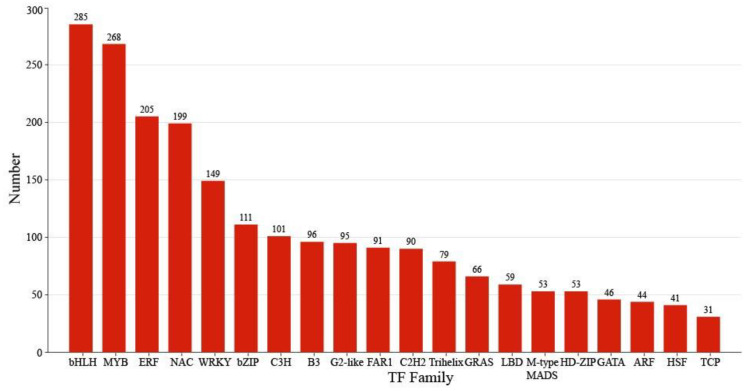
Classification of transcription factors.

**Figure 5:**
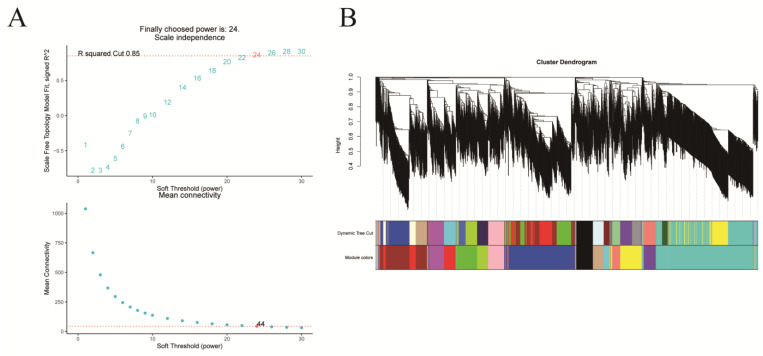

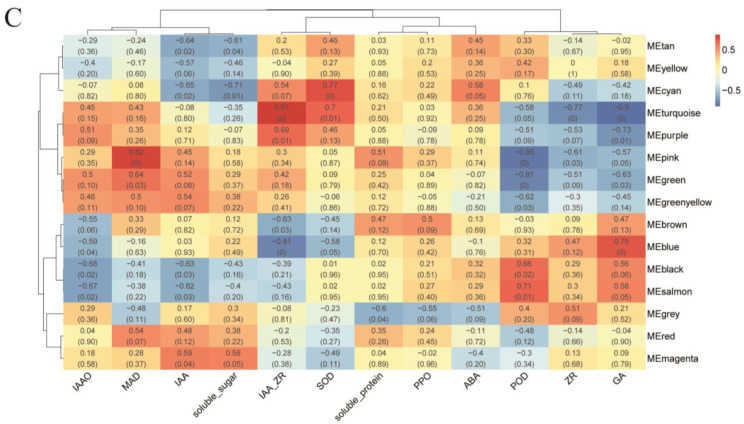
Weighted gene co-expression network analysis (WGCNA) of in vitro regeneration in *C. parthenoxylon*. (**A**) Determination of soft-thresholding power. (**B**) Module identification and clustering. (**C**) Module-trait correlation heatmap.

**Figure 6:**
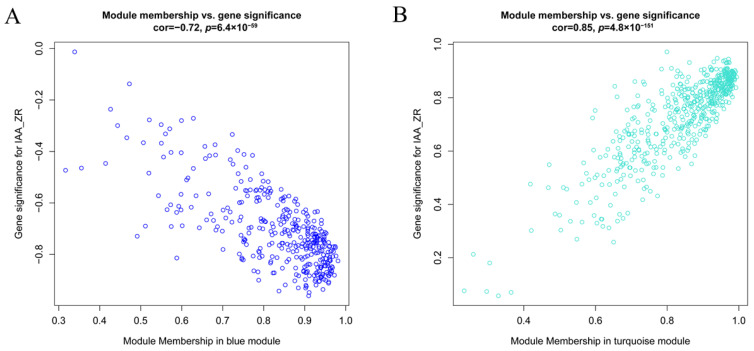

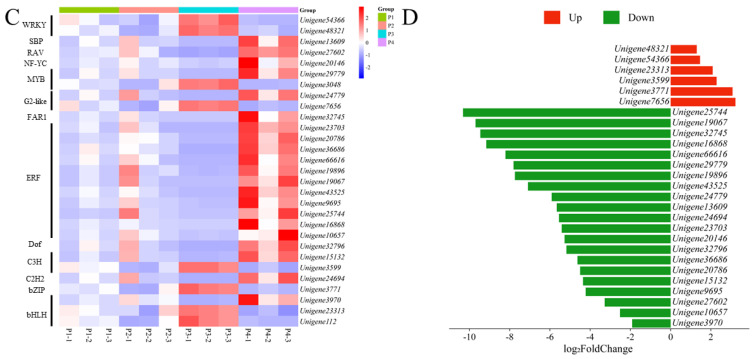
Functional characterization of key gene modules in *C. parthenoxylon* regeneration. (**A**) Green module gene significance (GS) vs. module membership (MM) scatter plot. (**B**) Turquoise module GS vs. MM scatter plot. (**C**) Heatmap of module-associated TFs. (**D**) Bar plot showing fold change of candidate TFs.

Comprehensive analysis identified 2162 transcription factors among the differentially expressed genes, with the notable enrichment of bHLH, MYB, ERF, and NAC family members during the in vitro regeneration of *C. parthenoxylon* (Figure 4). A weighted gene co-expression network analysis (WGCNA) of all DEGs and physiological parameters identified 15 distinct modules, among which the turquoise module showed a strong positive correlation with the IAA/ZR ratio, but negative correlations with ZR and GA levels, while the blue module was positively correlated with both IAA/ZR and GA (Figure 5C). Screening for hub transcription factors using stringent criteria (MM > 0.9 and |GS| > 0.85) identified 29 candidates, 27 of which were differentially expressed in the P3C vs. P3 comparison (Figure 6A,B). A total of 13 gene families were annotated. The ERF family was the most significantly enriched, containing 11 genes. Enrichment was also observed in other families such as bHLH, WRKY, and MYB (Figure 6C). Notably, several key regulators, including *Unigene48321* (WRKY), *Unigene54366* (WRKY), *Unigene23313* (bHLH), *Unigene3599* (C3H), *Unigene3771* (bZIP), and *Unigene7656* (G2-like), exhibited marked upregulation during pluripotent callus formation, with expression peaking at the P3 stage (Figure 6C,D).

### 2.5. Gene Expression Patterns Throughout the Efficient Regeneration of Adventitious Bud via K-Means Analysis

**Figure 7:**
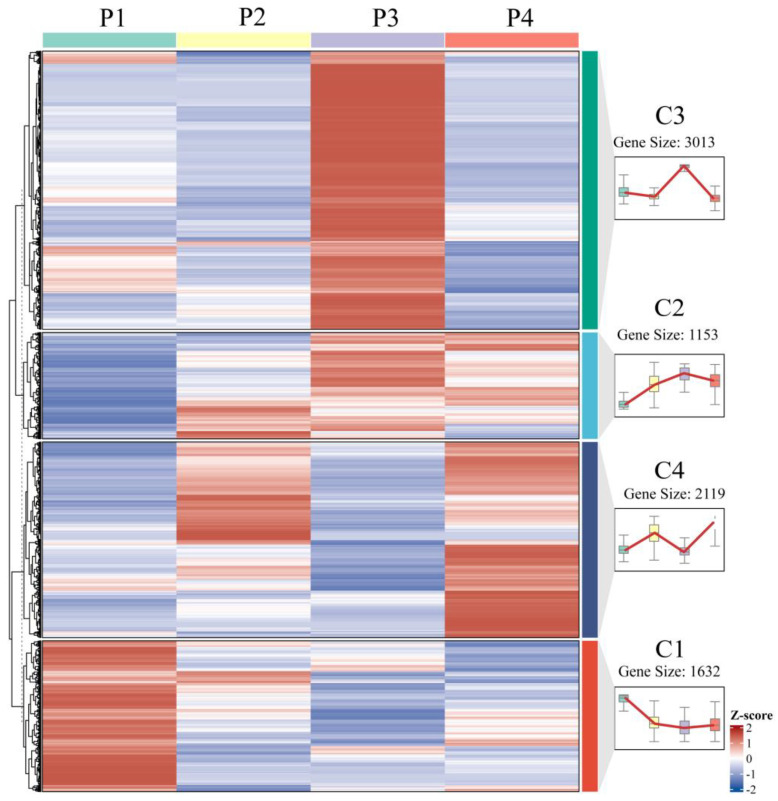
Expression dynamics of DEGs at various stages of in vitro regeneration.

Optimized by the elbow method, K-means clustering analysis categorized the 7917 differentially expressed genes (DEGs) identified during in vitro regeneration into four distinct clusters, each exhibiting a characteristic expression pattern (Figure 7). Cluster C1 genes displayed high expression levels in pre-inoculation samples (P1) followed by rapid downregulation during callus formation and subsequent stabilization. In contrast, cluster C2 genes exhibited their lowest expression at P1, gradually increased during callus formation and pluripotency acquisition stages, peaked at P3, and then declined during adventitious bud formation. Cluster C3 genes showed specific upregulation during callus proliferation with relatively low expression at other developmental stages, while cluster C4 genes demonstrated bimodal expression peaks during both callus formation and shoot initiation phases.

**Figure 8:**
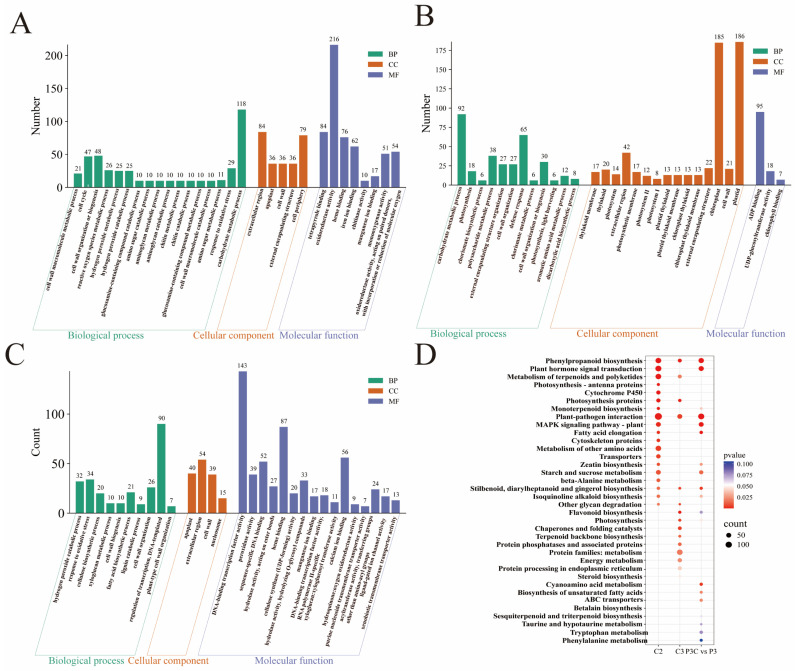
Functional enrichment analysis of DEGs. (**A**–**C**) Bar chart of GO enrichment analysis for DEGs. (**A**) C2 cluster. (**B**) C3 cluster. (**C**) P3C vs. P3. (**D**) Bubble plot of KEGG enrichment analysis for DEGs.

Focusing on the critical stage of pluripotency acquisition, comprehensive functional annotation of the highly expressed genes in clusters C2 and C3 was conducted using GO and KEGG enrichment analyses (Figure 8). The C2 cluster genes were significantly enriched in the carbohydrate metabolism, cell wall component and H_2_O_2_ metabolic processes, and redox enzyme activity regulation (Figure 8A). The C3 cluster genes primarily participated in carbohydrate and cell wall component metabolism, defense responses, and ADP binding, with notable enrichment in chloroplasts and plastids (Figure 8B). KEGG pathway analysis further revealed that C2 cluster genes were most abundantly represented in plant–pathogen interaction pathways, followed by phenylpropanoid biosynthesis, plant hormone signal transduction, and terpenoid/polyketide metabolism. The C3 cluster genes showed predominant enrichment in protein metabolism pathways, with a secondary representation in plant–pathogen interactions (Figure 8D).

The additional enrichment analysis of P3C vs. P3 DEGs demonstrated their primary involvement in transcriptional regulation, the oxidative stress response, and cell wall component metabolism processes, with cellular localization in the apoplast, extracellular region, and cell wall. Their molecular functions were associated with DNA-binding transcription factor activity and heme binding, and they were significantly enriched in pathways including the plant–pathogen interaction, phenylpropanoid biosynthesis, and plant hormone signal transduction (Figure 8C,D). Integrative analysis revealed that the DEGs collectively contribute to pluripotency acquisition by coordinately regulating cell wall component metabolism, phenylpropanoid biosynthesis, and plant hormone signal transduction pathways. This finding underscores the complex molecular network that governs cellular reprogramming during in vitro regeneration.

### 2.6. DEGs Related to Hormone Signal Transduction

**Figure 9:**
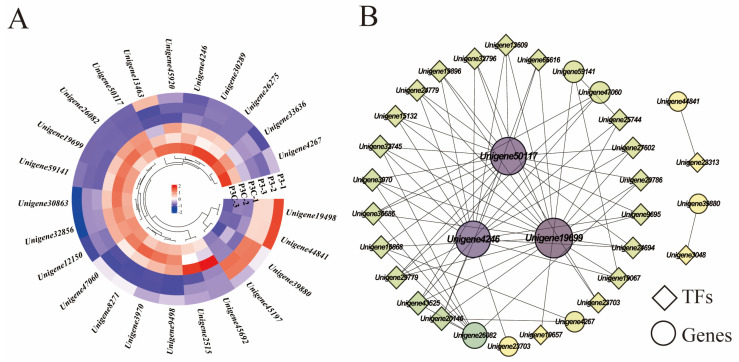
Hormone signal transduction pathways. (**A**) Gene expression heatmap. (**B**) Gene co-expression network. Each dot represents a gene and each line represents the regulatory relationship between genes: the darker and larger the dot, the higher the connectivity of the gene.

KEGG pathway analysis revealed the significant enrichment of plant hormone signaling transduction pathways during pluripotent callus acquisition, including auxin, CTK, ABA, JA, ETL, and GA signaling components. Five genes (*Unigene19699*, *Unigene32856*, *Unigene45197*, *Unigene44841*, and *Unigene45920*) exhibited consistently high expression during in vitro regeneration. Comparative transcriptomics revealed four upregulated genes in a pluripotent callus (P3) versus ordinary callus (P3C), encoding an ethylene-responsive transcription factor 1B homolog (*Unigene19498*), two auxin response factors (*Unigene12150* and *Unigene44841*), and an auxin-induced protein (*Unigene39880*). In contrast, twenty genes showed downregulation, particularly those involved in CTK, ABA, and JA signaling pathways (Figure 9A). Notably, auxin signaling transduction genes displayed divergent expression patterns, with *Unigene45197* and *Unigene32856* showing opposite trends. A correlation network analysis identified three key hormone-related genes, *Unigene50117* (encoding putative GH3.1 auxin-amido synthetase), *Unigene19699* (encoding TIFY 10A protein), and *Unigene4246* (encoding ARR2-like response regulator), as highly connected hubs. These genes likely mediate crosstalk between hormone signaling and transcriptional regulation during cellular reprogramming (Figure 9B).

### 2.7. DEGs Related to Cell Wall Metabolism

**Figure 10:**
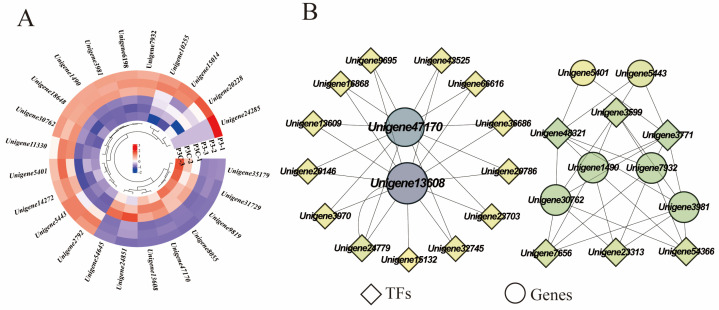
Cell wall metabolism. (**A**) Gene expression heatmap. (**B**) Gene co-expression network. Each dot represents a gene and each line represents the regulatory relationship between genes; the darker and larger the dot, the higher the connectivity of the gene.

During the acquisition of callus pluripotency in *C. parthenoxylon*, 23 genes were annotated to be involved in cell wall component metabolic processes, encoding important proteins such as expansins, pectin esterases, and xyloglucan endotransglycosylases/hydrolases (XTH). Among them, four genes encoding XTH (*Unigene8055*, *Unigene47170*, *Unigene13608*, and *Unigene54645*) exhibited relatively high expression levels during in vitro regeneration in *C. parthenoxylon*. Compared with ordinary calluses (P3C), all genes encoding pectin esterases, cellulose synthases, and UDP-arabinopyranose mutases in pluripotent calluses were upregulated in pluripotent calluses (P3). For genes encoding XTH and expansins, some were upregulated while others were downregulated (Figure 10A). Correlation analysis between genes related to cell wall metabolism and hub TFs revealed that two genes encoding XTH (*Unigene47170* and *Unigene13608*) were highly correlated with more transcription factors, suggesting that these genes may play important roles in the in vitro regeneration of *C. parthenoxylon* by regulating changes in cell wall components (Figure 10B). In addition, transcription factors WRKY (*Unigene48321*), C3H (*Unigene3599*), and bZIP (*Unigene3771*) may be involved in regulating the synthesis of cellulose and expansins.

## 3. Discussion

A robust in vitro regeneration system serves as the foundation for genetic transformation studies, involving intricate physiological and biochemical processes regulated by diverse nutrients, enzymes, and genes. The identification of key functional genes in this process not only provides critical genetic resources for deciphering the molecular mechanisms of bud differentiation in *C. parthenoxylon*, but also establishes a solid foundation for advancing molecular biology research in this species. These findings establish favorable conditions for future genetic engineering and breeding practices, greatly enhancing the capacity for targeted trait improvements in *C. parthenoxylon*.

### 3.1. Histological Features of Explant Regeneration

The in vitro regeneration process of *C. parthenoxylon* is accompanied by dynamic changes at both morphological and histological levels, which can be divided into three stages: callus formation, callus pluripotency acquisition, and adventitious bud formation. Among these, callus formation serves as the fundamental basis for the entire process. The formation of calluses from explants on a callus induction medium is a critical step where somatic cells gain totipotency and achieve the de novo regeneration of adventitious buds or roots, holding significant value in plant development research and crop genetic improvement [17]. A callus is an irregularly structured mass of cells containing a highly heterogeneous cell population [18]. Employing single-cell sequencing technology, Xu et al. [9] classified the callus derived from *Arabidopsis* hypocotyls into three distinct layers, among which the middle cell layer, with characteristics of a root apical quiescent center, exhibits an organ regeneration capacity. In this study, significant differences were observed between calluses formed under different induction conditions. Under the combined treatment of 6-BA and 2,4-D, distinct zones of actively dividing cells were observed within the callus. These zones were absent in the control group, indicating a strong correlation between the differentiation capacity of the callus and the presence of such proliferative regions. A similar phenomenon has been reported in both embryonic and non-embryonic callus tissues of cucumber [19]. This result indicates that the emergence of this zone is a characteristic manifestation of calluses acquiring pluripotency and a key factor for adventitious bud formation. In the study of leaf petiole regeneration in *Capsicum annuum*, the results indicated that disordered and dense meristematic cell clusters are crucial for root primordium formation [20]. Previous studies have confirmed that the larger the actively dividing cell zone in the callus, the stronger its pluripotency and the higher the frequency of bud regeneration [21]. Exogenous growth regulators, particularly auxin-like substances, play a major regulatory role in the formation and differentiation of *C. parthenoxylon* calluses. Studies have shown that different plant growth regulator combinations significantly influence the organogenesis induction efficiency in explants of the same genotype in *Capsicum annuum*. Among them, ZI treatment was more effective in inducing the formation of meristematic cell clusters and bud primordia in the epidermal and parenchyma tissues between the vascular bundles and epidermis. In contrast, 6-BA treatment tended to promote continuous cell division, leading to the formation of larger callus masses [20]. The present study revealed that 2,4-D exerts a crucial promotive effect during the acquisition of callus pluripotency, whereas NAA failed to induce a comparable response. This functional discrepancy may be attributed to their distinct regulatory pathways influencing endogenous auxin biosynthesis and local hormonal homeostasis, thereby modulating the in vitro regeneration process in *C. parthenoxylon* [22]. In this study, histological observations were conducted on the in vitro regeneration process of *C. parthenoxylon* under different exogenous hormone treatments. The similarities and differences in organogenesis were systematically compared, providing a reference for the selection of hormonal regimens and the evaluation of induction efficiency in vitro.

### 3.2. Dynamics of Endogenous Nutrients, Hormone Levels, and Enzyme Activities During In Vitro Regeneration

In this study, soluble sugars and soluble proteins exhibited inconsistent changes across different stages, with these differences resulting from the combined effects of cellular metabolic demands and regulatory mechanisms. Beyond serving as energy sources for plant life activities, soluble sugars can act as signaling molecules to regulate the activity and function of meristems [23]. For example, soluble sugars can influence the callus regeneration capacity by modulating plant hormone signals and reactive oxygen species (ROS) levels [24]. Peroxidase (POD), a cell-wall-localized protein that regulates lignin biosynthesis, is critically involved in processes related to cell wall loosening and rigidification [25,26]. Increased lignin synthesis leads to lignin accumulation in calluses and promotes browning, thereby exerting a negative regulatory effect on callus regeneration [27]. In this study, POD activity showed a continuous decline during callus formation and pluripotency acquisition, suggesting that POD may participate in *C. parthenoxylon in vitro* regeneration by regulating lignin synthesis and the cell wall [28]. Additionally, POD activity in pluripotent calluses was lower than in the control group, indicating that low POD activity facilitates the acquisition of callus pluripotency. The quantity and type of endogenous hormones are crucial for plant growth and development. The in vitro regeneration process relies on the synergistic action of multiple hormones, with auxins and cytokinins playing particularly critical roles [29]. As a core hormone, auxin functions prominently in plant organ morphogenesis [30]. Studies have shown that high auxin concentrations promote callus formation but inhibit its differentiation [31], which may explain the initial increase followed by a decrease in endogenous auxin during *C. parthenoxylon* in vitro regeneration. In this study, the zeatin riboside (ZR) content increased during callus formation and then gradually decreased, a trend consistent with the dynamic changes in ZR observed during the in vitro regeneration of pepper [20]. Previous research has demonstrated that gibberellin (GA) levels decline rapidly during callus pluripotency acquisition, but rise sharply during adventitious bud formation, indicating that GA inhibits pluripotency acquisition while promoting adventitious bud formation [32]. Another study noted that adding GA biosynthesis inhibitors can upregulate *LBD16* expression [33] and *LBD16* is involved in acquiring cellular pluripotency in calluses [8]. Furthermore, a comparative analysis of callus tissues with varying differentiation capacities indicated that elevated concentrations of ZR and ABA, in conjunction with reduced levels of IAA and GA, promote the acquisition of pluripotency in calluses. Studies have shown that the content of zeatin (ZT) in embryonic calluses derived from cucumber cotyledons is higher than that in non-embryonic calluses [19]. These results indicate that both the local hormonal balance and the type of auxin source are regulated by exogenous growth regulators and play a critical role in plant in vitro regeneration [22]. Notably, ABA acts as a downstream signaling mediator of 2,4-D and auxin enhances plant cell totipotency by modulating ABA biosynthesis, signal transduction, and transcriptional regulation [34]. Therefore, the subsequent studies will focus on the differences in endogenous hormone levels among callus tissues with varying differentiation capacities. By exogenously applying plant hormones or hormone synthesis inhibitors during the in vitro regeneration process, particularly at the stage of pluripotency acquisition, we aim to enhance the pluripotent state of callus tissues and even induce pluripotency in ordinary calluses, thereby improving the overall regeneration efficiency.

### 3.3. Effects of Transcription Factors on Regeneration

TFs are a class of protein molecules that can specifically bind to specific sequences in the upstream region of the 5′ end of genes, ensuring that target genes are expressed with a specific intensity in a spatiotemporally specific manner. Accumulating evidence indicates that transcription factors play important roles in plant in vitro regeneration [9,35]. For instance, MYB transcription factors, as one of the largest families of plant TFs, play key roles in the cell cycle, root growth, and organ development [36]. During the development of soybean calluses, the expression of numerous genes encoding bHLH and MYB transcription factors undergoes significant changes [37], which is consistent with the results of this study, suggesting that these transcription factors may be crucial for the formation and differentiation of a *C. parthenoxylon* callus. Through WGCNA, this study identified several TFs closely related to auxin and cytokinin. Among them, *Unigene48321* (encoding WRKY), *Unigene54366* (encoding WRKY), *Unigene23313* (encoding bHLH), *Unigene3599* (encoding C3H), *Unigene3771* (encoding bZIP), and *Unigene7656* (encoding G2-like) may be involved in the acquisition of *C. parthenoxylon* callus pluripotency. The expression levels of these genes were significantly upregulated during the acquisition of callus pluripotency and reached a peak. A large number of studies have confirmed that different TFs participate in the acquisition of callus pluripotency through various ways. WRKY23, bHLH041, and bZIP are involved in auxin-induced callus formation. Among them, WRKY23 and bHLH041 synergistically establish callus pluripotency by promoting and inhibiting the transcription of root stem cell factors *PLT1*, *PLT2*, and *WOX5*, respectively [38], while bZIP promotes callus formation by forming a complex with LBD [39]. In addition, in maize, calluses overexpressing the *G2* gene turns green earlier and has more green spots; this gene promotes callus differentiation by upregulating the expression of genes related to chloroplast development [40]. Studies have shown that the *C3H* gene contains hormone-related cis-acting elements and is up-regulated during the transition from a non-embryogenic to embryogenic callus in *Dimocarpus longan*, suggesting its functional involvement in the acquisition of callus pluripotency [41].

### 3.4. Transcriptome Pathways in Regeneration of C. parthenoxylon

Clustering and enrichment analyses of DEGs during *C. parthenoxylon* in vitro regeneration revealed that plant hormone signal transduction and cell wall component metabolism play important roles in this process, particularly during the stage of callus pluripotency acquisition. Among the genes involved in plant hormone signal transduction, *Unigene50117* (encoding putative indole-3-acetic acid-amido synthetase GH3.1), *Unigene19699* (encoding protein TIFY 10A), and *Unigene4246* (encoding two-component response regulator ARR2-like protein isoform X2) may play major roles in the acquisition of callus pluripotency. The GH3.1 regulates hormone homeostasis by catalyzing the conjugation of free auxin with amino acids, and its overexpression inhibits auxin signaling [42]. In this study, the expression level of *GH3.1* in pluripotent calluses was significantly lower than that in ordinary calluses, suggesting that it may be a suppressor of callus pluripotency acquisition [43]. Additionally, TIFY 10A may also act as a suppressor in this process. As a negative regulator of the jasmonic acid (JA) signaling pathway, TIFY 10A is not only induced by JA, but also functions as an early auxin response factor, with its auxin-induced expression being independent of the JA signaling pathway [44]. Previous studies have found that calluses formed from hypocotyl explants pretreated with JA exhibits a significantly increased rate of new bud regeneration [45]. The ARR2 belongs to the type-B response regulators. The regulation of *WUS* by type-B ARRs is crucial for maintaining stem cells in the shoot apical meristem [46]. However, type-B ARR family members exhibit multiple functions and antagonistic relationships in in vitro shoot regeneration [47], suggesting that ARR2 may also act as a suppressor of pluripotency acquisition in *C. parthenoxylon* calluses.

Cell wall remodeling involves dynamic changes in cell wall components and plays an important role in plant in vitro regeneration [48]. Studies have shown that treating explants with pectinase and cellulase can lead to the moderate degradation of the cell wall, which significantly promotes callus formation in poplar explants [49]. Xyloglucan endotransglycosylase/hydrolase can modify the structure of cellulose–xyloglucan complexes in cell walls by breaking and reconnecting xyloglucan chains, thereby realizing cell wall reconstruction [50]. The results of this study showed that two genes encoding XTH, *Unigene43170* and *Unigene13608*, are involved in the acquisition of the pluripotency of *C. parthenoxylon* callus. Another study confirmed that the gene encoding cell wall relaxation enzyme XTH9 is only expressed in cells around progenitor cells, thereby causing different mechanical stresses in these cells, and ultimately activating cell polarity in progenitor cells to promote the formation of meristems [51]. In addition, this study also found that the transcription factors WRKY (*Unigene48321*), C3H (*Unigene3599*), and bZIP (*Unigene3771*) may be involved in the regulation of cellulose and expansin synthesis. Studies have shown that the gene *FAD-BD* encoding the BBE-like enzyme is a target of the AtbZIP59-LBD16 complex, and this enzyme is involved in regulating lignin monomer metabolism and cell wall integrity [39,52].

## 4. Materials and Methods

### 4.1. Plant Materials and Culture Conditions

Stem segments and internodes from tissue-cultured seedlings of *C. parthenoxylon* were used as explants. Callus induction was conducted by inoculating explants into two callus induction media (CIM): the treatment medium (MS + 1.0 mg/L 6-BA + 0.2 mg/L 2,4-D) and the control medium (MS + 1.0 mg/L 6-BA + 0.2 mg/L NAA). To sustain continuous callus growth and acquisition of pluripotency, the callus induction medium was replaced every 25 days. Two subculture cycles were performed over the total 50-day cultivation period. Subsequently, the induced calluses were transferred to adventitious shoot induction medium (MS + 1.0 mg/L 6-BA + 0.8 mg/L TDZ + 0.05 mg/L NAA) to induce adventitious shoots. All culture media contained 30 g/L sucrose and 5.0 g/L carrageenan, with the pH adjusted to 5.6–5.8. The cultures were grown at 25 ± 2 °C under a 14 h photoperiod and a light intensity of 2500 lux.

### 4.2. Morphological and Anatomical Observations During Regeneration

Observations were systematically carried out every 5 days after inoculation until adventitious buds had fully formed. Morphological changes throughout callus differentiation, such as treatment duration and features of bud formation, were documented. Histological examination was conducted using paraffin sectioning according to the protocol established by Bryant et al. [53].

### 4.3. Sampling Method

Based on morpho-anatomical observations during regeneration, samples were systematically collected at 0 days (P1: before inoculation), 20 days (P2: swelling at both ends of the stem to form a large amount of callus tissue), 35 days (P3: callus tissue proliferates and turns yellow), and 60 days (P4: adventitious shoot formation), and numbered P1, P2, P3, and P4 in sequence. A parallel control sample (P3C) was collected at 35 days from cultures maintained under control conditions for comparative analysis with P3. Sampling consisted of collecting entire stem segments at P1, calluses from the swollen ends of the segments at P2, and material from the callus surface at the P3, P3C, and P4 stages. After sampling, the samples were wrapped in tin foil, snap-frozen in liquid nitrogen for 5 min, and then stored at −80 °C for subsequent determinations of physiological indices and transcriptome sequencing. Three biological replicates were set for each group.

### 4.4. Determination of Endogenous Nutrients, Hormone Content, and Enzyme Activity

All indicators were measured following the instructions of the respective kits. Specifically, soluble sugar content was determined using Plant Soluble Sugar Content Assay Kit (BC0030, Solarbio, Beijing, China). Soluble protein content was measured with the Plant Soluble Protein ELISA Kit (SP29721, Saipei biotechnology, Wuhan, China). Malondialdehyde (MDA) content was assayed using MDA Assay Kit (BC0020, Solarbio, Beijing, China). Polyphenol oxidase (PPO) activity was detected using PPO Assay Kit (BC0190, Solarbio, Beijing, China). Peroxidase (POD) activity was measured with POD Activity Detection Kit (BC5190, Solarbio, Beijing, China). Superoxide dismutase (SOD) activity was assayed using the SOD Activity Detection Kit (BC0170, Solarbio, Beijing, China). Indoleacetic acid oxidase (IAAO) activity was determined using IAAO Activity Assay Kit (AKPL022C, BOXBIO, Beijing, China). For enzyme activity, U/(g·min) was specifically defined as the amount of enzyme that causes an increase or decrease in absorbance (OD) of 0.01 per minute per gram of fresh tissue under the specified assay conditions. This unit represents the catalytic efficiency, relative enzyme concentration in the tissue, and the reaction rate. Endogenous hormone contents (IAA, GA, ABA, and ZR) were determined by high-performance liquid chromatography–tandem mass spectrometry (HPLC-MS/MS) as described by Li et al. [20]. Three biological replicates were included per group, with duplicate technical measurements performed for each biological replicate.

### 4.5. RNA Library Construction and Sequencing

Total RNA was extracted using R6827 Plant RNA Kit (R6827-00, Omega Bio-Tek, Guangzhou, China). RNA quality was evaluated using NanoDrop One spectrophotometer (Wilmington, DE, USA) and Qubit 3.0 Fluorometer (Carlsbad, CA, USA), and further verified by agarose gel electrophoresis. The quality control assessment included examinations for contamination, RNA fragment size distribution, and RNA purity. The mRNA was enriched using oligo (dT) magnetic beads. The purified mRNA was then fragmented by incubation with a fragmentation reagent. Using the fragmented mRNA as template, first-strand cDNA was synthesized, followed by second-strand cDNA synthesis according to the manufacturer’s protocol. The double-stranded cDNA was subsequently subjected to end repair, adenylation (A-tailing), and adapter ligation. The resulting products were purified, PCR-amplified, and circularized to finalize the library construction. Sequencing library was constructed with the MGIEasy RNA Library Prep Kit (1000006383, MGI, Shenzhen, China). After passing library quality inspection, sequencing was performed on the BGI high-throughput sequencing platform DNBSEQ-T7 (MGI, Shenzhen, China).

### 4.6. Differential Gene Screening and Enrichment Analysis

Raw image data files from high-throughput sequencing were subjected to transformation, filtering by fastp (0.21.0, default) [54], concatenation by Trinity (2012.11.0, –min_kmer cov_2) [55], and clustering by cd-hit (4.8.1) [56] to generate unigene sequences. Reads containing over 5% N bases, low-quality reads, adapter-contaminated reads, and PCR duplicates were removed during filtering. The TransDecoder (5.5.0, –m 50) was employed to predict the coding regions of Unigenes. Due to the paired-end sequencing capability of the DNBSEQ-T7 (MGI, Shenzhen, China), the expression level of each unigene in the samples was quantified using fragments per kilobase of transcript per million mapped reads (FPKM). Differentially expressed genes (DEGs) were screened under the criteria of |log_2_(fold change)| > 1 and *p*-value < 0.05, with the latter considered indicative of significant enrichment. The protein sequences encoded by Unigenes were aligned against existing protein databases (data accessed on 3 July 2025), including UniProt and Nr, as well as the metabolic pathway database KEGG, using diamond blastp (2.0.6.144, –evalue 1 × 10^−5^) [57]. This analysis provided functional annotations of the sequences and identified potential metabolic pathways in which the proteins may be involved.

### 4.7. Transcription Factor (TF) Analysis and Gene Expression Classification

The predicted protein sequences of DEGs were compared against the corresponding transcription factor database (PlantTFDB) [58] using HMMER’s hmmscan tool to obtain transcription factor-related information (data accessed on 3 July 2025). Genes with similar expression patterns among DEGs were clustered into modules using a weighted calculation method, followed by analysis of the correlation between these modules and physiological and biochemical indices. Modules significantly associated with external traits were regarded as key modules and the genes within them were considered to be related to these external traits. To screen for key TFs regulating the in vitro regeneration of *C. parthenoxylon*, the correlation coefficient (MM) between genes and modules in the key modules was calculated, along with the significance of gene significance (GS) between genes and corresponding traits, and hub TFs were subsequently identified [59]. To investigate the expression patterns of differentially expressed genes (DEGs) across different developmental stages, the K-means clustering algorithm was applied to categorize the expressed genes into four distinct clusters [60]. Functional enrichment analyses, including Gene Ontology (GO) and Kyoto Encyclopedia of Genes and Genomes (KEGG) pathway analyses, were subsequently performed on the genes within each cluster.

### 4.8. Quantitative Real-Time PCR Validation

To validate the reliability of transcriptome sequencing results, 12 DEGs were randomly selected to perform quantitative real-time PCR (qRT-PCR). Gene-specific primers were designed (sequences provided in Table S1) with Actin serving as the internal reference gene for normalization. Total RNA was reverse-transcribed using the PrimeScript™ RT reagent kit (RR037A, TaKaRa, Osaka, Japan), followed by PCR amplification using TB Green Premix Ex Taq™ II (RR820A, TaKaRa, Japan) under the following conditions: initial denaturation at 95 °C for 1 min, followed by 40 cycles of 95 °C for 15 s, 60 °C for 15 s, and 72 °C for 30 s. Relative gene expression levels were calculated using the 2^−ΔΔCT^ method [61], with three biological replicates analyzed for each sample.

### 4.9. Statistical Analysis

All experimental data were processed and analyzed using established statistical software. Raw data were organized and pre-processed using Microsoft Excel 2010. Statistical analyses were performed with SPSS 26.0, including one-way ANOVA followed by LSD post hoc tests for multiple comparisons, with statistical significance set at *p* < 0.05. Physiological parameters and qRT-PCR data are presented as mean ± standard deviation (SD) and visualized using GraphPad Prism 8.4.3. The GO and KEGG enrichment analysis of DEGs was conducted in Tbtools-II [62]. Transcriptomic data visualization was conducted using R 4.5.0. Specifically, volcano plots were generated with the ggplot2 package, Venn diagrams with the ggVennDiagram package, and heatmaps with the pheatmap package. Weighted gene co-expression network analysis (WGCNA) was also carried out using the corresponding the WGCNA package. Additionally, the K-means clustering for gene expression classification was performed using an online analysis platform in https://www.bioinformatics.com.cn (accessed on 10 July 2025) [63], and the specific approach is described by Yu et al. [60]. The gene co-expression networks were constructed and visualized using Cytoscape 3.10.0.

## 5. Conclusions

This study systematically investigated the key mechanisms governing in vitro regeneration in *C. parthenoxylon*, revealing a strong dependence of the regeneration process on callus pluripotency. Actively dividing cell zones within callus tissue function as pivotal sites for the acquisition of pluripotency and subsequent adventitious bud formation. The size of these zones may be used as an indicator to evaluate the effectiveness of induction conditions and the differentiation capacity of the callus. At the physiology level, the high ZR and ABA levels combined with low IAA and GA concentrations and lower POD activity may be more conducive to the acquisition of callus pluripotency. At the transcription factor level, the genes associated with plant hormone signal transduction, such as *GH3.1*, *TIFY10A*, and *ARR2*, as well as those involved in cell wall metabolism, including *XTH*, are closely correlated with auxin- and cytokinin-regulated TFs and play essential roles in the acquisition of pluripotency and regeneration. Together, these findings provide a comprehensive characterization of the histological features, physiological dynamics, and molecular networks governing in vitro regeneration in *C. parthenoxylon*. This study establishes a critical foundation for elucidating the molecular mechanisms of bud differentiation. In subsequent studies, we will systematically optimize the in vitro regeneration culture conditions for *C. parthenoxylon* to enhance the pluripotency of the callus. Furthermore, key genes identified in earlier screening will be genetically transformed into degenerated pluripotent calluses and even conventional callus tissues, with the aim of improving their pluripotency. This work will help establish a stable and efficient genetic transformation platform for *C. parthenoxylon*.

## Data Availability

The raw sequence data reported in this paper have been deposited in the Genome Sequence Archive (Genomics, Proteomics & Bioinformatics 2021) in the National Genomics Data Center (Nucleic Acids Res 2025), China National Center for Bioinformation/Beijing Institute of Genomics, and Chinese Academy of Sciences (GSA: CRA019608) that are publicly accessible at https://ngdc.cncb.ac.cn/gsa (accessed on 10 October 2024).

## Supplementary Materials

The following supporting information can be downloaded at: https://www.mdpi.com/article/10.3390/plants14192945/s1, Figure S1. Validation of Gene Expression. Data are presented as mean ± SD (*n* = 6). Figure S2. Venn Diagram of Group P3C vs. P3 and Other Groups. Figure S3. The photos of *C. parthenoxylon*. A: Superior clones of *C. parthenoxylon*. B: Tissue-cultured seedlings of *C. parthenoxylon*. Figure S4. The experimental methodology flowchart. Table S1. List of primers; Table S2. Matrix of pairwise Pearson correlation coefficients between samples; Table S3. List of differentially expressed genes (DEGs) in the plant hormone signaling pathway during in vitro regeneration; Table S4. List of differentially expressed genes (DEGs) in the cell wall pathway during in vitro regeneration.

## Abbreviations

The following abbreviations are used in this manuscript:

POD	Peroxidase
IAAO	Indole-3-acetic acid oxidase
PPO	Polyphenol oxidase
MDA	Malondialdehyde
SOD	Linear dichroism
IAA	Indole-3-acetic acid
ZR	Zeatin riboside
GA	Gibberellins
ABA	Abscisic acid
GO	Gene Ontology
KEGG	Kyoto Encyclopedia of Genes and Genomes
WGCNA	Weighted gene co-expression network analysis
DEGs	Differentially expressed genes
