# Supplementary material for: Integrated Multi-Omics Reveals Mechanism of Adventitious Buds Regeneration in In Vitro Cultures of Cinnamomum parthenoxylon
Source: Plants (Basel). 2025 Sep 23;14(19):2945. doi: 10.3390/plants14192945 (PMC12525742; doi:10.3390/plants14192945)
Supplement: Supplementary file 1 [file plants-14-02945-s001.zip › plants-3863299-Supplementary Material.pdf]

Table S1 List of primers

Unigenes ID	Sequence ID	Primer ID	Primer Sequence(5’-3’)
Unigene40538	*CpActin*	CpActin-F	CTGCATGTCTTCACATGATCAGAA
		CpActin-R	GAGCCTCCAATCCAGACACTGTAC
Unigene19885	*CpSPS*	CpSPS-F	TGGTTACTCTTCCGATGCAGT
		CpSPS-R	TGACGTAAGAAGTGCAGGTCA
Unigene11953	*CpPPO*	CpPPO-F	CGACTCGGTGTTGAAGGTGA
		CpPPO-R	GCTTCTTGTGGTCCTCGACA
Unigene1273	*CpSOD1*	CpSOD1-F	TCCTGAGGATGAAAACCGCC
		CpSOD1-R	CCCTTCCCAAGATCATCGGG
Unigene994	*CpRBOHD*	CpRBOHD-F	TTCCTCTCCCATTTCCACGC
		CpRBOHD-R	GTTTGATTGAGGGGGAGGGG
Unigene10445	*CpCKX*	CpCKX-F	GGCCCATATGGTCGATCAGG
		CpCKX-R	CTGCGATTTGAGACCTGGGT
Unigene12854	*CpGA2OX*	CpGA2OX-F	ACTACCCTCCATGCCCAGAT
		CpGA2OX-R	TCGCCAACGATGACGAAGAA
Unigene3655	*CpARF*	CpARF-F	GCCTACGCCAACTCAAGAGT
		CpARF-R	CAAAGGCATCACCAGCAACC
Unigene14524	*CpA-ARR*	CpA-ARR-F	ACCATGTCACAGCTGTGGAT
		CpA-ARR-R	CCAGTCATTCCGGGCATACA
Unigene12150	*CpDELLA*	CpDELLA-F	CTCTTCTCCCCTGCTCAAGC
		CpDELLA-R	GAGACCGTGCATGAGGTTGA
Unigene19498	*CpERF1/2*	CpERF1/2-F	GCCGCTTTTGCTATACGTGG
		CpERF1/2-R	TTACCGCTCGCACTTTTCCT
Unigene11161	*CpGH3*	CpGH3-F	CATCCGATTCCTCCAGCTCC
		CpGH3-R	TCCCTTCCAAATTCTCGCCC
Unigene850	*Cp4CL*	Cp4CL-F	CCCTCTCCACTCCTACTGCT
		Cp4CL-R	GGGGAGTTGGAGAGGAGGAT

Table S2. Matrix of pairwise Pearson correlation coefficients between samples

ID	P1-1	P1-2	P1-3	P2-1	P2-2	P2-3	P3-1	P3-2	P3-3	P3C-1	P3C-2	P3C-3	P4-1	P4-2	P4-3
**P1-1**	1.00	0.94	0.93	0.90	0.87	0.93	0.89	0.89	0.89	0.91	0.90	0.90	0.90	0.92	0.89
**P1-2**	0.94	1.00	0.94	0.90	0.88	0.92	0.88	0.88	0.88	0.92	0.91	0.91	0.91	0.93	0.91
**P1-3**	0.93	0.94	1.00	0.89	0.89	0.93	0.88	0.89	0.89	0.92	0.91	0.90	0.91	0.93	0.91
**P2-1**	0.90	0.90	0.89	1.00	0.89	0.88	0.86	0.86	0.86	0.92	0.93	0.93	0.90	0.93	0.92
**P2-2**	0.87	0.88	0.89	0.89	1.00	0.87	0.85	0.85	0.85	0.93	0.92	0.94	0.91	0.93	0.94
**P2-3**	0.93	0.92	0.93	0.88	0.87	1.00	0.89	0.89	0.89	0.90	0.90	0.89	0.90	0.90	0.89
**P3-1**	0.89	0.88	0.88	0.86	0.85	0.89	1.00	0.93	0.92	0.88	0.87	0.87	0.86	0.88	0.87
**P3-2**	0.89	0.88	0.89	0.86	0.85	0.89	0.93	1.00	0.93	0.88	0.88	0.87	0.86	0.89	0.87
**P3-3**	0.89	0.88	0.89	0.86	0.85	0.89	0.92	0.93	1.00	0.88	0.87	0.87	0.86	0.89	0.87
**P3C-1**	0.91	0.92	0.92	0.92	0.93	0.90	0.88	0.88	0.88	1.00	0.95	0.95	0.94	0.95	0.95
**P3C-2**	0.90	0.91	0.91	0.93	0.92	0.90	0.87	0.88	0.87	0.95	1.00	0.96	0.93	0.94	0.95
**P3C-3**	0.90	0.91	0.90	0.93	0.94	0.89	0.87	0.87	0.87	0.95	0.96	1.00	0.93	0.95	0.96
**P4-1**	0.90	0.91	0.91	0.90	0.91	0.90	0.86	0.86	0.86	0.94	0.93	0.93	1.00	0.94	0.94

**P4-2**	0.92	0.93	0.93	0.93	0.93	0.90	0.88	0.89	0.89	0.95	0.94	0.95	0.94	1.00	0.95
**P4-3**	0.89	0.91	0.91	0.92	0.94	0.89	0.87	0.87	0.87	0.95	0.95	0.96	0.94	0.95	1.00

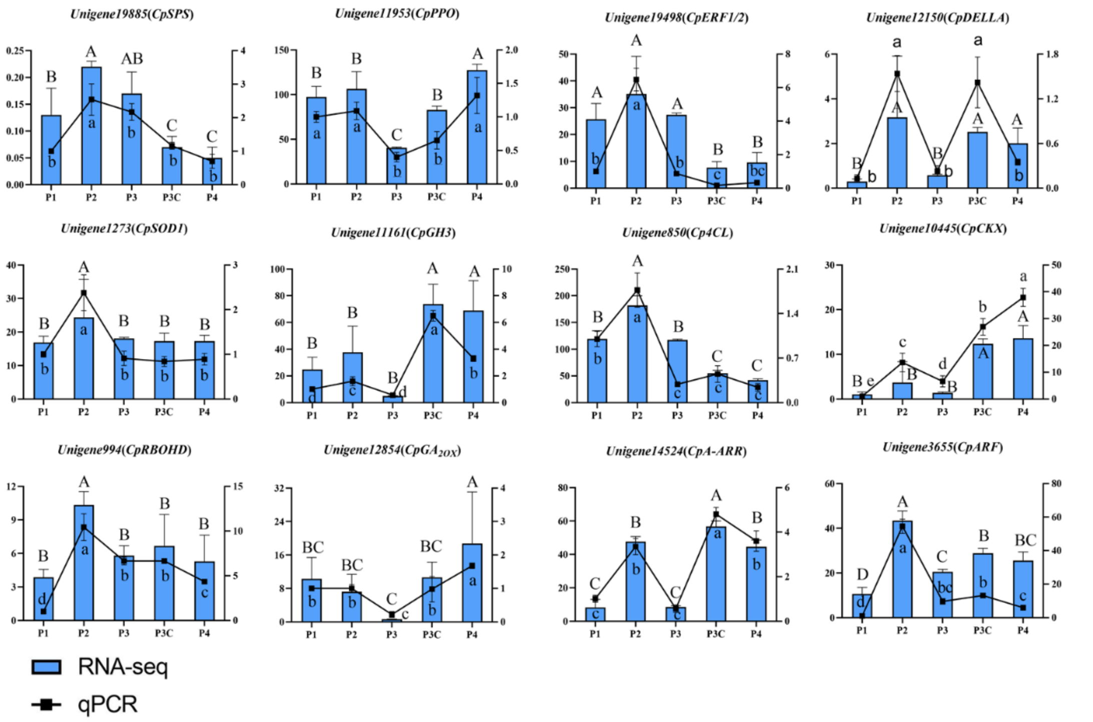

Figure S1. Validation of Gene Expression. Data are presented as mean ± SD (n = 6). Different uppercase (A-E) and lowercase (a-e) letters indicate statistically significant differences ($p < 0.05$) among groups for the RNA-seq and qRT-PCR results, respectively, based on a one-way ANOVA with an LSD multiple comparison test.

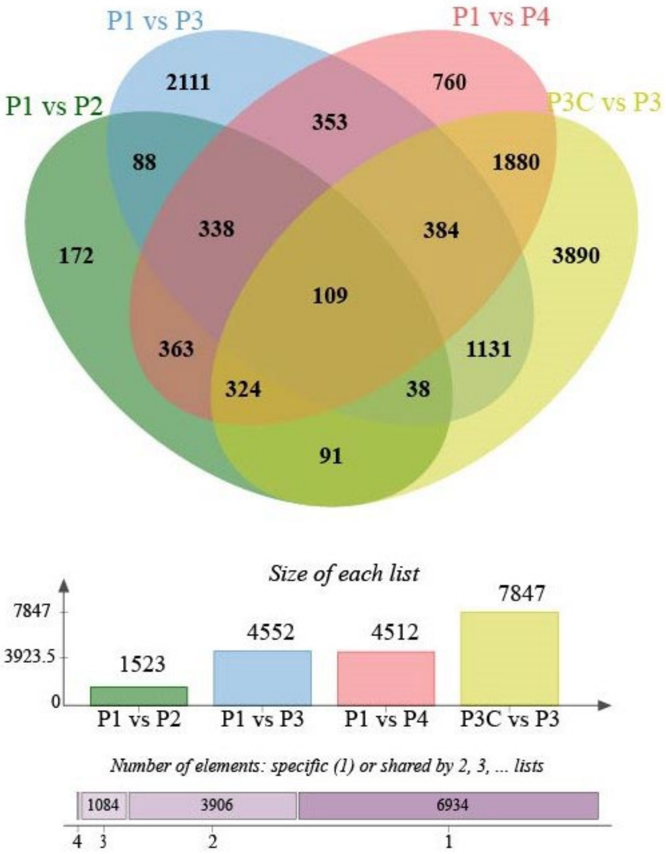

Figure S2. Venn Diagram of Group P3C vs P3 and Other Groups

Table S3 List of differentially expressed genes (DEGs) in the plant hormone signaling pathway during *in vitro* regeneration

Hormone	Gene ID	Description	FPKM			
			P1	P2	P3	P4
ABA	*Unigene30289*	Abscisic acid receptor PYL2	0.99	9.30	0.28	12.27
ABA	*Unigene8271*	Protein-serine/threonine phosphatase	7.30	5.97	2.07	8.02
ABA	*Unigene59141*	Protein-serine/threonine phosphatase	3.48	10.92	0.37	11.92
CTK	*Unigene45920*	Histidine-containing phosphotransfer protein 1 isoform X1	38.93	66.01	32.28	82.28
CTK	*Unigene33636*	Signal transduction response regulator	1.07	8.97	4.02	10.31
CTK	*Unigene13463*	Two-component response regulator	0.20	0.77	0.68	0.72
CTK	*Unigene4267*	Two-component response regulator ARR2-like protein isoform X2	0.40	0.57	0.19	1.40
CTK	*Unigene26275*	Two-component response regulator ORR3-like protein	0.00	8.55	0.00	5.34
ETL	*Unigene19498*	Ethylene-responsive transcription factor 1B-like protein	25.68	23.65	27.34	9.55
GA	*Unigene12150*	DELLA protein GAI-like protein	0.23	3.17	0.58	2.01
IAA	*Unigene39880*	Auxin response factor	2.08	2.80	7.96	3.55
IAA	*Unigene44841*	Auxin-induced protein	58.22	61.40	71.12	20.81
IAA	*Unigene45692*	Auxin-induced protein 6B-like protein	0.97	9.05	0.28	10.37
IAA	*Unigene45197*	Auxin-responsive family protein	21.65	27.95	115.04	21.79
IAA	*Unigene9498*	Auxin-responsive protein	41.09	38.06	15.90	61.64
IAA	*Unigene32856*	Auxin-responsive protein	98.10	158.12	171.06	350.40

IAA	*Unigene26082*	Auxin-responsive protein	4.21	11.06	2.67	34.74
IAA	*Unigene30863*	Auxin-responsive protein SAUR36-like protein	0.89	2.37	1.03	2.48
IAA	*Unigene47060*	GH3 auxin-responsive promoter	12.79	10.35	2.79	20.38
IAA	*Unigene2515*	Indole-3-acetic acid-amido synthetase GH3.9	1.76	1.31	1.09	2.73
IAA	*Unigene50117*	Putative indole-3-acetic acid-amido synthetase GH3.1	6.96	7.99	2.29	28.35
JA	*Unigene19699*	Protein TIFY 10A	26.67	58.80	9.16	128.53
JA	*Unigene4246*	Protein TIFY 9	0.28	0.65	0.22	1.27
JA	*Unigene3970*	Transcription factor MYC	11.38	15.78	5.62	30.08

Table S4 List of differentially expressed genes (DEGs) in the cell wall pathway during *in vitro* regeneration

Gene ID	Description	FPKM			
		P1	P2	P3	P4
Unigene1490	Cellulose synthase	16.27	11.85	41.06	16.32
Unigene35179	Expansin	1.87	22.28	8.26	29.91
Unigene31729	Expansin	1.24	14.87	1.62	12.12
Unigene18648	Expansin	9.71	12.24	20.86	11.05
Unigene14272	Pectin acetylesterase	19.48	28.34	49.27	21.61
Unigene20228	Pectinesterase	4.34	3.77	8.94	5.03
Unigene6198	Pectinesterase	3.29	4.29	11.00	5.41
Unigene10255	Pectinesterase	2.14	1.28	2.04	0.76
Unigene30762	Pectinesterase	21.29	10.81	44.75	11.99
Unigene11330	Pectinesterase	0.47	1.08	3.13	1.04
Unigene5401	Pectinesterase	0.10	0.24	1.15	0.22
Unigene7932	Pectinesterase	0.21	0.02	1.19	0.10
Unigene3981	Pectinesterase	1.08	0.41	3.20	0.19
Unigene9819	Putative polygalacturonase	1.47	2.50	0.87	3.42
Unigene2792	Putative polygalacturonase	1.44	1.18	1.38	0.51
Unigene24285	UDP-arabinopyranose mutase	0.00	0.00	7.03	0.00
Unigene15014	Xyloglucan endotransglucosylase/hydrolase	3.10	1.00	2.20	2.59
Unigene24851	Xyloglucan endotransglucosylase/hydrolase	16.27	4.42	0.69	2.74
Unigene8055	Xyloglucan endotransglucosylase/hydrolase	49.51	106.69	83.73	181.91
Unigene47170	Xyloglucan endotransglucosylase/hydrolase	138.89	43.62	38.30	562.58
Unigene13608	Xyloglucan endotransglucosylase/hydrolase	63.52	31.56	22.95	249.81
Unigene54645	Xyloglucan endotransglucosylase/hydrolase	296.11	56.54	24.29	233.24
Unigene5443	Xyloglucan endotransglucosylase/hydrolase	0.22	0.32	1.30	0.20

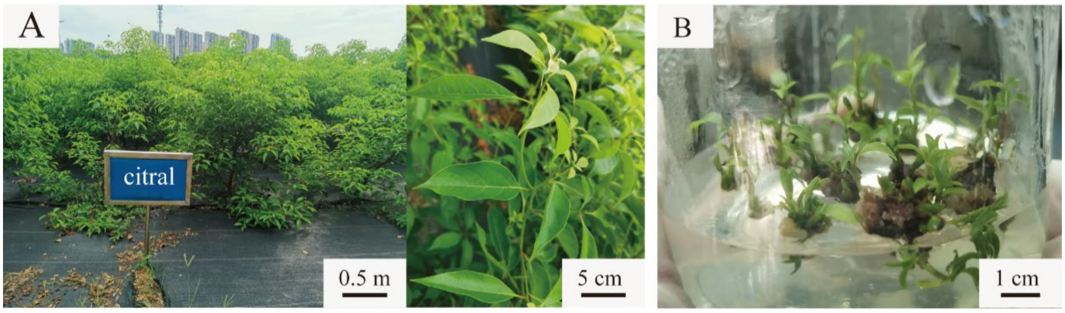

Figure S3. The photos of *C. parthenoxylon*. A: Superior clones of *C. parthenoxylon*. B: Tissue-cultured seedlings of *C. parthenoxylon*.

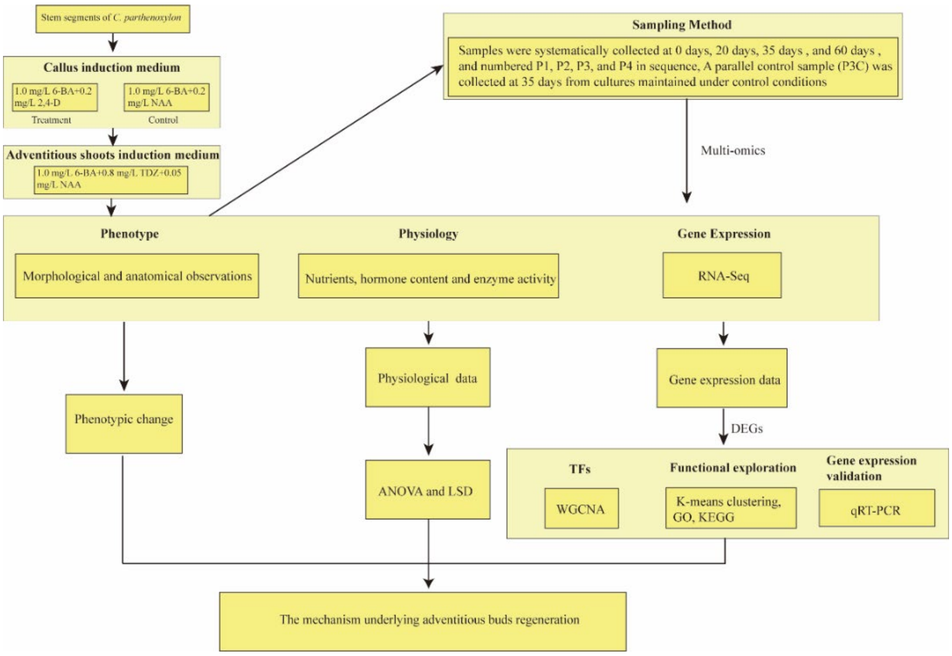

Figure S4. The experimental methodology flowchart.
